# A Place to Locate

**Published:** 1885-11

**Authors:** 


### A Place to Locate.

According to a report made to a Georgia State Medical Society, there are in that State three towns with a population of over 1,000 each, having no physician. Evelyn, population 1,500, Aerial, population 1,200, and Fouche, population 1,000, are the towns referred to. While there is on an average one physician to 600 people in the United States, Macon, Ga., with a population of 23,000, has but twenty-four physicians, and Savannah, with a population of 41,000, has only thirty-five doctors.

---

Dr. Morrell advises that every physician keep an antidote bag, which should contain every drug and instrument needed in ordinary cases of poisoning. It should always be kept filled and ready for use, so that, in case of emergency, the doctor could take it along or send for it, and not be compelled to look for stray bottles or instruments at a time when a life may depend upon a minute.—*Boston Medical and Surgical Journal.*

---

According to *Daniel's Medical Journal*, Austin, Tex., has been afflicted with a severe epidemic of dengue, or break-bone fever. Dr. Daniel states that the disease is now on a rapid decline, simply for want of material, all, or nearly all, big and little, old and young, male and female, rich and poor, having had it.

